# Pathophysiological Roles of Histamine Receptors in Cancer Progression: Implications and Perspectives as Potential Molecular Targets

**DOI:** 10.3390/biom11081232

**Published:** 2021-08-18

**Authors:** Phuong Linh Nguyen, Jungsook Cho

**Affiliations:** College of Pharmacy and Integrated Research Institute for Drug Development, Dongguk University-Seoul, Goyang, Gyeonggi 10326, Korea; phuonglinh212126@gmail.com

**Keywords:** histamine, histamine receptors, histamine receptor ligands, molecular target, tumor microenvironment, cancer progression, prognostic biomarkers

## Abstract

High levels of histamine and histamine receptors (HRs), including H1R~H4R, are found in many different types of tumor cells and cells in the tumor microenvironment, suggesting their involvement in tumor progression. This review summarizes the latest evidence demonstrating the pathophysiological roles of histamine and its cognate receptors in cancer biology. We also discuss the novel therapeutic approaches of selective HR ligands and their potential prognostic values in cancer treatment. Briefly, histamine is highly implicated in cancer development, growth, and metastasis through interactions with distinct HRs. It also regulates the infiltration of immune cells into the tumor sites, exerting an immunomodulatory function. Moreover, the effects of various HR ligands, including H1R antagonists, H2R antagonists, and H4R agonists, on tumor progression in many different cancer types are described. Interestingly, the expression levels of HR subtypes may serve as prognostic biomarkers in several cancers. Taken together, HRs are promising targets for cancer treatment, and HR ligands may offer novel therapeutic potential, alone or in combination with conventional therapy. However, due to the complexity of the pathophysiological roles of histamine and HRs in cancer biology, further studies are warranted before HR ligands can be introduced into clinical settings.

## 1. Introduction

The incidence of cancer and resultant mortality are swiftly growing worldwide [1]. A plethora of literature has illustrated that genetic and epigenetic alterations are associated with multi-step processes in cancer development and progression, transforming normal cells into malignant derivatives [2,3]. Advanced understanding of cancer biology and the molecular basis of therapeutic responses would contribute to deriving novel potential targets to achieve maximal efficacy with reduced side effects in cancer therapy [4]. Recently, intense attention has been paid to the tumor microenvironment (TME), the environment around a tumor. The TME consists of a complex mixture of extracellular matrix and non-transformed cells, such as immune cells, endothelial cells, and stromal cells. While tumor cells can influence the microenvironment by releasing extracellular signaling molecules, the cells in the TME also constantly interact with tumor cells, affecting tumor evolution and growth, invasion, and metastasis [5,6]. Therefore, microenvironmental regulation of tumor progression may be an effective strategy for treating cancer [5,6,7].

Histamine is one of the main mediators involved in inflammatory and immune responses. The major cellular sources of histamine are the mast cells (MCs), basophils, histaminergic neurons, and the gastric enterochoromaffin-like cells (ECLs), while minor quantities are also released from epithelial cells or T cells [8,9,10]. In addition, high levels of histamine and histamine receptors (HRs) in a wide range of different types of cancer indicate their involvement in the complex biology of cancer [11,12]. In fact, the expression of HRs in a variety of human cancer cell lines has supported the role of histamine as an autocrine growth factor that increases the cell proliferation rate [13,14,15,16]. Moreover, histamine has been shown to stimulate diverse carcinogenesis-associated events, such as cell invasion, migration, and angiogenesis, demonstrating its crucial role in cancer progression [17,18,19,20]. Several HR antagonists and inhibitors of histamine synthesis or histamine transporters have been reported to show preventive effects on cancer cell growth [19,21].

Cancer is a complex and highly heterogeneous disease. Despite the advances in cancer research over the last decades, the outcomes of cancer therapy are not fully satisfactory. In order to overcome this fatal disease and to improve the therapeutic efficacy, better understanding of the different cellular and molecular participants in the tumor and TME and identification of novel potential molecular targets is necessary. In this review, we aim to summarize the pathophysiological roles of histamine and the complex networks of HRs in cancer progression. In addition, we provide recent advanced knowledge concerning the pharmacological effects of selective HR ligands and their potential prognostic values in cancer treatment. This review will explore novel molecular targets with potential benefits that can be used alone or in combination with traditional therapy for the treatment of various types of cancer.

## 2. Histamine and Histamine Receptors

Histamine is an endogenous biogenic monoamine that acts as a neurotransmitter in the nervous system or as a local mediator in the gastrointestinal tract, skin, and immune system [22]. Histamine is distributed ubiquitously throughout the body, mediating numerous pathophysiological conditions [23]. Histamine is synthesized from the essential amino acid histidine exclusively by histidine decarboxylase (HDC), which catalyzes the decarboxylation reaction. After being synthesized, it is stored at high concentrations within cytoplasmic granules or the vesicles of cells such as MCs, basophils, ECLs, and nerve cells [22,24].

**Figure 1:**
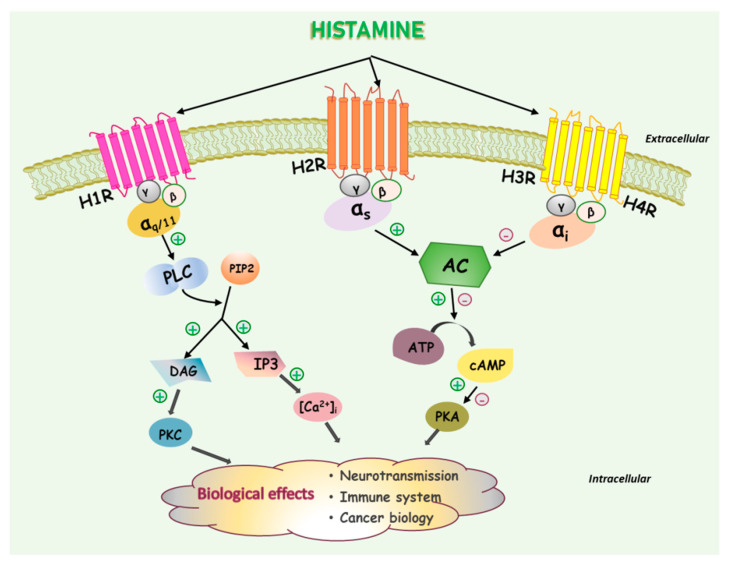
Signaling pathways mediating the biological effects of the histamine receptor subtypes. Each receptor is activated by endogenous histamine. The biological effects depend on the HR subtypes and cell types. H1R: histamine H1 receptor; H2R: histamine H2 receptor; H3R: histamine H3 receptor; H4R: histamine H4 receptor; PLC: phospholipase C; PIP2: phosphatidylinositol 4,5-bisphosphate; DAG: diacylglycerol; IP3: inositol 1,4,5-trisphosphate; [Ca^2+^]_i_: intracellular calcium; PKC: protein kinase C; PKA: protein kinase A.

Histamine exerts a wide variety of biological actions through interactions with four subtypes, which belong to the G protein-coupled receptor (GPCR) group: histamine H1 receptor (H1R), H2R, H3R, and H4R [22,23]. The molecular biology and pharmacology of these receptors have been the subject of numerous reviews and books [22,23,24,25]. Depending on the characteristics of the receptor subtypes to which histamine is paired and the intracellular signaling pathways coupled, the resulting physiological responses may be different. H1R was the first discovered. It couples to Gα_q/11_ proteins, which activates the PLC/IP_3_/DAG/protein kinase C signaling pathway and subsequently increases the intracellular Ca^2+^ levels (Figure 1). Its functions in allergy-related conditions are well elucidated [8,26]. H2R is coupled to Gα_s_ proteins and triggers the release of cAMP, mediating gastric acid secretion from parietal cells in the stomach [24]. In contrast, both H3R and H4R bind to Gα_i_ proteins and inhibit cAMP production. H3R is almost exclusively expressed in the nervous system, mediating the actions of histamine as a neurotransmitter. Considerable attention has been paid to H3R as a potential drug target for the treatment of several brain disorders, including Alzheimer’s and Parkinson’s diseases, cerebral ischemia, and sleep/wake disorders [27,28,29]. Finally, H4R is primarily expressed in hematopoietic cells, indicating its function in immunomodulation. Indeed, it has been strongly suggested that H4R is involved in allergies, inflammation, autoimmune disorders, and possibly cancer [28,30]. The development of selective ligands for H3R and H4R may facilitate the elucidation of their physiological and pathological roles in the nervous system and immune system, respectively. In addition to H3R, H4R has also attracted attention as a novel target to modulate various histamine-mediated immunologic and inflammatory disorders [28,30]. The signaling pathways mediating the biological effects of the respective histamine receptor subtype are depicted in Figure 1.

The H1R subtype is ubiquitously distributed, with predominant expression on MCs, smooth muscle, and endothelial cells. H2R is mainly expressed on gastric parietal cells. However, it is also expressed on smooth muscle and endothelial cells. H3R is primarily located in the central nervous system (CNS), particularly on histaminergic neurons. While H4R expression on neurons is controversial [31], it is mainly expressed on hematopoietic cells, including leukocytes, eosinophils, and monocytes [9,10]. Surprisingly, all four subtypes of HR are expressed on human cancer cells. Therefore, elucidation of the pathophysiological roles of histamine and HR subtypes in various types of cancer cells is worthwhile. A wide variety of agonists and antagonists specifically acting on their respective receptor subtypes have been utilized to investigate their pharmacological and clinical significance in cancer therapy. The distribution of HR subtypes and their representative specific agonists and antagonists/inverse agonists are listed in Table 1.

## 3. Effects of Histamine on the Immune Cells in a Tumor Microenvironment (TME)

The causal concatenation between cancer and inflammation is comprehensively accepted in that the hallmark of inflammation-associated cancer is the existence of inflammatory mediators and immune cells in the TME [38,39]. The TME is comprised of stromal cells as well as different types of immune cells, such as MCs, macrophages (MOs), NK cells, T and B cells, and myeloid-derived suppressor cells (MDSCs) [38]. The MCs and basophils are the main sources of histamine [10], and distinct immunoregulatory effects of histamine are exhibited through its binding to cognate receptors on these cells [9]. Here we describe how the histamine system recruits key immune cells into the TME and regulates the infiltrated cells to exert their effects on tumor progression.

### 3.1. Effects of Histamine on Mast Cells

Accumulating evidence has suggested that histamine is one of the most potent inflammatory mediators, intensifying and prolonging the inflammatory responses [34,40,41]. The majority of granule-stored histamine in normal tissues is found in MCs, a type of innate immune cells derived from myeloid stem cells [42,43,44]. MCs have important roles in many physiological and pathological immune responses as well as in many inflammatory diseases [45,46,47,48,49]. In addition, many studies have demonstrated that MCs are commonly found in various tumors and can even infiltrate into the tumor sites. The infiltrated MCs express inflammatory phenotypes, which respond to chemotactic stimuli, resulting in cell migration to inflamed or damaged tissues [44,49].

One of the possible mechanisms mediating the actions of MCs and their mediators on tumor progression is through stimulation of angiogenesis by releasing pro-angiogenic enzymes and factors. The pro-angiogenic mediators include histamine, vascular endothelial growth factor (VEGF), tumor necrosis factor-α (TNF-α), interleukin (IL)-6, and IL-8 [50,51,52]. In contrast, however, it has been demonstrated that MCs can also affect the antitumor immunity via activation of various types of immune cells such as NK cells, dendritic cells (DCs), cytotoxic T (CD8+) cells, and T-helper (CD4+) cells at tumor sites [53]. Therefore, it is still highly controversial whether MCs and their mediators are pro-tumorigenic or antitumorigenic [44,49,54]. With the double-edged characteristics of MCs, their pathophysiological roles may be dependent on the types of cancer and other factors mediating tumor progression [11,44,49].

Among the various mediators released from MCs, histamine may play a key role in chemotaxis to recruit MCs to the TME through interactions with HRs. MCs derived from H4R-deficient mice were found to lose their capability to migrate, indicating that the chemotactic response of these cells to histamine is mediated by H4R [55]. Recruitment of MCs to inflamed sites amplifies the inflammatory reactions mediated by histamine and may favor the establishment of chronic inflammatory responses [9]. Ghosh et al. reported that HDC-deficient mice showed defective angiogenesis with low levels of VEGF in inflammatory tissues [56]. Meanwhile, MC-deficient mice showed no significant differences in their angiogenesis and VEGF levels due to the infiltrated HDC-producing non-MCs, such as MOs. These findings suggest that histamine derived from either MCs or non-MCs plays a significant role in angiogenesis.

### 3.2. Effects of Histamine on Antigen-Presenting Cells

Antigen-presenting cells (APCs) incorporate MOs, DCs, and B cells. APCs interact with T cells to initiate adaptive immune responses by exhibiting invading pathogens (antigens) bound by major histocompatibility complex (MHC) proteins on their surfaces. Innate immunity is also maintained in part by APCs. By displaying tumorigenic antigens, APCs can directly influence the differentiation and activation of T cells. By this mechanism, tumorigenic antigens can be eliminated from organisms [57].

In early studies, MOs were frequently discovered within human cancers and in experimental cancer cell lines, acting as professional APCs. In addition, MOs are crucial cellular sources of cytokines and chemokines. It was found that MOs in the tumor or in the peripheral blood restrict the antitumor activity of NK cells by affecting cell proliferation and cytokine gene transcription. As a result, NK cells cannot effectively respond to IL-2 or interferon-α (IFN-α), a potent activator of NK cells [58,59,60]. The mechanism underlying the inhibition of NK cells by MOs involves the formation of reactive oxygen species (ROS), particularly hydrogen peroxide, by the action of NADPH oxidase in monocytes [60]. Thus, finding a way to abrogate the MO-induced inhibition of NK cells can provide beneficial advantages to regenerate the function of the immune system to defeat cancer.

Histamine has been demonstrated to possess favorable properties that can reverse the inhibition of NK cells by MOs, through H2R-mediated inhibition of NADPH oxidase activity [60]. In addition, the action of histamine is synergistic with IL-2 and IFN-α, leading to the killing of a large number of NK cell-sensitive human tumor cells in vitro and in vivo [58,59,60,61,62]. Moreover, histamine has been reported to regulate antioxidant enzymes, which subsequently leads to increased ROS production, thereby inhibiting the proliferation of myeloid leukemia, malignant melanoma (MM), and renal cell carcinoma [63,64]. Besides, in addition to maintaining the potent antitumor properties of IL-2, histamine was found to constrain tumor growth in a rat model of prostate adenocarcinoma [65]. Furthermore, clinical data advocate for the co-administration of histamine with IL-2 and/or IFN-α to prolong survival time and to suppress tumors, such as MM and liver melanoma, which are refractory to IL-2 or IFN-α therapy [66]. It is worth highlighting the use of histamine in combination with low-dose IL-2 in a Phase IV trial to prevent the relapse of acute myeloid leukemia and improve overall survival (OS) [66,67]. These findings strengthen the importance of combination therapy in cancer treatment and encourage the investigation of histamine as an adjuvant for cancer immunotherapy.

DCs are considered as a potent APC population regulating adaptive immune responses due to their necessity for T cell-mediated cancer immunity [68]. It is generally thought that immature DCs do not appropriately activate T cells, which may lead to immune tolerance [69]. Conversely, mature DCs capture antigens; move to lymph nodes; and present them to activate T cells, including CD4+ T helper (TH1 and TH2) cells, CD8+ cytotoxic T lymphocytes (CTLs), and regulator T (Treg) cells [70]. Histamine in the TME has diverse effects on tumor immunology through maturation of DCs. On the one hand, the activation of H1R and H3R in the differentiation process of DCs leads to positive stimulation of the tumor-related antigen-presentation capacity, the production of pro-inflammatory cytokines (IL-12, IFN-γ), and TH1 polarization [71,72]. On the other hand, H2R activation suppresses the ability of antigen presentation and enhances IL-10 production and TH2 polarization [72,73,74]. H4R expressed on DCs decreases the secretion of CCL2 and IL-12 and limits the ability of DCs to induce TH2 responses [75,76].

B cells, as one of the APCs regulating T cells in immune responses, are capable of triggering anticancer properties [77,78]. B cells express HRs and also secrete histamine [33]; however, there is no evidence showing a correlation between histamine and the APC function of B cells. B cells have an antibody-producing capacity against tumor antigens to directly kill tumor cells [79]. Histamine induces the anti-IgM-stimulated proliferation of B cells, which is reversed in H1R-knockout (KO) mice. Besides, the function of B cell-producing antibodies against T cell-independent antigens is abolished in H1R-KO mice, suggesting an important role of H1R signaling in the antitumor responses of B cells [80]. It has been shown that histamine acts as an immunoregulatory factor, reducing immunoglobulin production in human B cell lines [81] and in immunized mice [82]. The suppression of immunoglobulin production by histamine is prevented by ranitidine, an H2R antagonist. Raniditine has been reported to inhibit tumor growth, which is B cell-dependent and associated with an enhanced B cell population and antitumor antibodies [83].

### 3.3. Effects of Histamine on T Cells

HRs are ubiquitously expressed on the surface of T cells (Table 1), including CD8+ CTLs and CD4+ T cells. CD4+ T cells are divided into TH1, TH2, and Treg cells. With regard to CD4+ T cells, H1R is expressed at relatively high levels in TH1 cells, while H2R presents preferentially in TH2 cells [84]. The regulation of specific T cells after antigen recognition via MHC has opposing effects on tumor cells. TH1 cells have powerful antitumor activity through activation of NK cells as well as CD8+ CTLs and increased expression of MHC and costimulatory molecules [85]. Conversely, TH2 cells suppress TH1 cells, prohibiting antitumor process [86,87]. Thus, histamine can possess either pro- or antitumor responses by targeting antigen-specific T cells, subsequently modifying the related antibody isotype responses.

In the previous part of this review, we presented the idea that MO-induced inhibition of cytokine activation could be restricted by histamine. MOs also intensely inhibit IL-2-induced expression of CD69, an activation antigen expressed on CD4+ and CD8+ T cells as well as on NK cells [88]. Therefore, histamine may indirectly optimize the activation of T cells. Indeed, histamine dihydrochloride exerting agonist activity on H2R showed a strong reduction in blood monocyte counts and an induction of melanoma-specific T cells, indicating leukemia- and melanoma-free survival, respectively [67]. Upon ensuring the rational T cell activity, histamine can act as a protective agent against T cell proliferation. A sizable fraction of CD4+ or CD8+ T cells acquired apoptotic morphology after interacting with MOs, and histamine almost completely prevented MO-induced cell death in all subsets of T cells [88].

However, histamine has been demonstrated to disrupt the balance between TH1 and TH2 and Treg in neoplastic tissues, leading to changes in cytokine secretion. For example, systemic histamine treatment decreases the expression of IFN-γ and IL-12 from TH1 but increases IL-10-secreting TH2 expression in colorectal tumor implants. Both IFN-γ and IL-12 are potent cytokines for antitumor immunity because they support cell-mediated immune responses [89,90], while IL-10 inhibits TH1 cell proliferation and downregulates cell-mediated immunity [91]. These findings suggest a stimulatory impact of histamine on tumor growth [92].

Treg cells, a specialized subpopulation of T cells, act to suppress the immune response through inhibition of T cell proliferation and cytokine production, thereby maintaining homeostasis and self-tolerance. Treg cells accumulated in the tumor tissues also mediate immunosuppression by constraining the activities of CD8+ CTLs and CD4+ T helper cells. Hence, this type of cell plays an important role in tumor development [85,93,94]. Histamine, acting through H2R, positively interferes with the peripheral antigen tolerance induced by Treg cells [95]. Foxp3 expression in Treg cells serves as a master regulator of Treg function and development. Interestingly, cimetidine, an H2R antagonist used to inhibit gastric acid secretion, has been reported to decrease the stability of the Foxp3 protein, resulting in consequent loss of Treg capacity and stimulation of cellular immunity [96,97,98].

CD8+ CTLs are known to contribute to the antitumor response due to the secretion of cytokines or cytotoxic molecules, ultimately leading to the apoptosis of tumor cells [53,85]. Several studies have agreed that the number of infiltrating CD8+ cells is conducive to a better response to chemotherapy [99]. Stimulation of human CD8+ CTLs in the presence of histamine causes a higher production of IL-16, a chemoattractant for T cells, which is blocked by the H2R antagonist [100]. IL-16 is also associated with the regulation of the growth and recruitment of CD4+ T cells during inflammatory responses and is considered to be a prognostic factor of OS for aggressive prostate cancer [100,101].

### 3.4. Effects of Histamine on Myeloid-Derived Suppressor Cells

MDSCs are a barrier to antitumor immune responses due to their function of inhibiting T cells, DCs, NK cells, and MOs, and inducing Treg cell development [102,103,104,105,106,107]. The expression of H1R~H3R and HDC in MDSCs has been documented [108,109], suggesting a contribution of histamine to modulate their activities. Apart from its influence on the survival and proliferation of MDSCs, histamine differentially regulates Arg1 and iNOS gene expression and enhances the levels of IL-4 and IL-13, resulting in MDSC-induced T cell suppression [108]. H2R has been particularly implicated in the regulation of MDSC activities by histamine. In a mouse model of lung cancer, cimetidine reversed the effects of histamine on MDSCs by increasing the apoptosis of MDSCs, reducing Arg1 and iNOS expression, and decreasing MDSC accumulation [110]. In another study, ranitidine also modified myeloid cell populations, resulting in the inhibition of breast tumor development and metastasis in mice [111]. However, HDC-KO mice showed a high rate of colon and skin carcinogenesis, mediated by reduced myeloid maturation and the accumulation of immature myeloid cells, indicating the crucial roles of HDC in myeloid cell differentiation in early cancer development [109].

**Figure 2:**
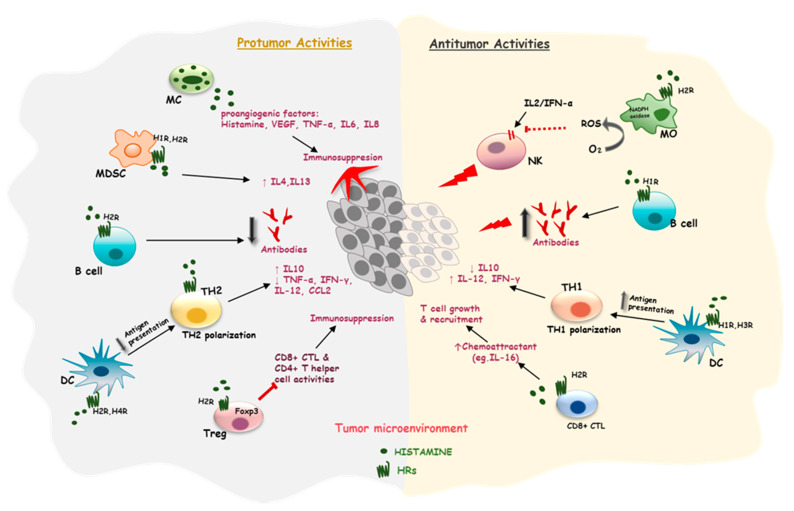
Bivalent roles of histamine and histamine receptor subtypes in the tumor microenvironment (TME). Histamine may exert either pro- or antitumor activities during tumor progression. Histamine also establishes cross-talk with other tumor-infiltrating immune cells in the TME by interacting with its corresponding receptors expressed on the cell surface, thus altering the final tumor outcomes. Representative actions of histamine in these cells are depicted in the figure. MC: mast cell; MDSC: myeloid-derived suppressor cell, TH1: CD4+ T helper 1 cell; TH2: CD4+ T helper 2 cell; CD8+ CTL: cytotoxic T lymphocyte; NK: natural killer; MO: macrophage; DC: dendritic cell; HRs: histamine receptors.

Taken together, histamine and HR subtypes may have a wider impact on the recruitment of various types of immune cells into the TME as well as on the regulation of their activities mediating tumor immunity. The multidimensional roles of the histamine system in regulating the immune cells in the TME, thereby exerting either pro- or antitumor activity, are delineated in Figure 2. This knowledge can spark new therapeutic solutions in tumor immunotherapy; however, the specific roles of histamine and its cognate receptors on immunomodulation should be fully clarified in future studies.

## 4. Pathophysiological Roles of Histamine and Histamine Receptors in Cancer and Their Implications as Potential Targets in Cancer Treatment

The effects of histamine on tumor biology depend on the interactions between histamine and its functional HRs on the surface of tumor cells or tumor-surrounding cells. In the previous section, we have summarized how histamine regulates the immune responses via immune cells in the TME and influences tumor development and growth. In this section, recent advances made in understanding the roles of histamine and its receptors on tumor cells are discussed. We also provide a number of potential therapeutic approaches targeting HR networks for cancer treatment.

### 4.1. Histamine in Cancer Progression

High concentrations of histamine have been observed in various human malignancies such as colorectal cancer (CRC) [112], breast cancer (BC) [113], and MM [17] as well as in many experimental carcinomas [18], suggesting the involvement of histamine in cancer biology. Based on immunohistochemical and reverse transcription-polymerase chain reaction studies, HDC activities are also high in some tumors, namely CRC, MM, and non-small cell lung cancer (NSCLC) [114,115,116]. In addition, the blood levels of histamine in patients with a newly diagnosed solid tumor are nearly three-fold greater than in healthy individuals or noncancerous disease controls. Following surgical removal of the malignancy, the level of histamine remains high for 2 months and then drops to the normal range by 3 months after surgery [117]. Collectively, these findings suggest that histamine synthesis is increased in the presence of a tumor.

During tumor progression, histamine has been implicated in a number of tumor cell activities, from the fundamental stages of tumor growth and invasion up to metastasis. However, its impact on tumor progression remains controversial as to whether histamine exerts detrimental or beneficial effects, facilitating cancer inhibition or promotion. Several studies have proven a strong correlation between upregulated HDC activity and a poor prognosis in different types of cancer. In human CRC, for example, increased proliferative activity and lymph node invasion were strongly correlated with a high density of HDC in the TME [118]. The effect of histamine released into the TME was also assessed on MM progression in a mouse model of primary skin tumors induced by cancerous B16-F10 MM cells. Markedly accelerated tumor growth and moderately increased metastatic colony-forming potential were reported, along with rising levels of local histamine production in the TME of mice with cells constitutively expressing the full-length HDC [119], confirming the involvement of histamine in the MM progression.

In contrast, however, there are considerable data indicating that histamine plays an effective role in the inhibition of carcinogenesis. Administration of a histamine-generating probiotic (hdc^+^ *Lactobacillus reuteri*) reduced the number and size of colon tumors in CRC and decreased the gene expression of pro-inflammatory mediators and cancer-related cytokines, while the HDC-deficient *L. reuteri* mutant did not suppress carcinogenesis [120]. In another study with oral squamous cell carcinomas (OSCC), using a hamster cheek pouch model, an injection of chlorin p6 (Cp6)-histamine conjugate enhanced the efficacy of photodynamic therapy (PDT) and resulted in the complete regression of large tumors. Based on this finding, conjugating histamine to Cp6 is proposed to be a promising approach to improve the efficacy of PDT for the treatment of head and neck cancer and OSCC [121]. Consistent with these observations, the administration of low doses of histamine (1 mg/kg body weight, s.c.) reduced the severity of boron neuron capture therapy-linked mucositis in the same experimental model when this therapy was applied to treat oral cancer [122]. Collectively, these results indicate that elevated levels of histamine and HDC activity exert significant roles in the suppression of inflammation as well as tumorigenesis in different types of cancer, including CRC and OSCC.

Thus, histamine can exert pro- or antitumor activity when it directly interacts with tumor cells. If the exact correlation between histamine and cancer progression is fully elucidated, the modulation of cellular pathways mediating the effect of histamine could be an excellent target as an adjuvant treatment along with typical cancer therapy. Therefore, placing more emphasis on research intended to fully understand the association of histamine and HRs with cancer progression is necessary. In the next section, we discuss the recent exploitation of HRs as a novel molecular target for cancer treatment by shedding new light on the roles of each HR subtype during tumor development.

### 4.2. Histamine Receptors in the Regulation of Cancer Progression and Their Implications as Potential Therapeutic Targets

#### 4.2.1. Histamine H1 Receptor

The expression of H1R has been observed in a variety of cancer cell lines and human neoplastic lesions such as CRC, hepatocellular carcinoma (HCC), OSCC, ovarian cancer (OC), BC, NSCLC, and MM [123]. Based on an integrative genomic analysis using the PrognoScan database [124], 23 out of 153 tests showed an association of microarray expression of the H1R gene with cancer prognosis [125]. Although the relationship between the expression of H1R and prognosis was found to vary in different types of cancers, several studies showing the correlation in certain cancer types have been published. Zhao et al. reported that H1R was frequently upregulated in HCC compared to normal tissues, which was significantly associated with both OS and recurrence-free survival in HCC patients [126]. To further confirm this observation, they performed other functional experiments to test the potential effects of H1R on tumor growth and metastasis. Both in vitro and in vivo studies showed that upregulation of H1R expression promoted HCC cell growth by inducing cell cycle progression and suppressing cell apoptosis [126]. Moreover, H1R also mediated the migration and invasion of HCC cells by stimulating the formation of lamellipodia and the production of matrix metalloproteinase 2 (MMP-2) through suppressing the activation of cAMP [126]. A cohort study measuring the H1R expression in patients with OSCC in association with clinicopathological prognostic factors showed similar results [127]. H1R was rarely expressed in OSCC, but its expression was significantly correlated with advanced tumor stages. Patients with H1R expression showed a markedly poor disease-free survival rate [127]. Additionally, by using bioinformatics tools to interrogate various published BC databases, H1R was found to be overexpressed in basal and HER2-enriched BC samples, which was significantly associated with a shorter OS and metastasis-free survival [128]. These data suggest that H1R in tumor cells may be a potential oncoprotein, and it is associated with a poor prognostic value in cancer.

Because overexpression and upregulation of H1R in some cancer types has been shown to positively influence tumor progression, researchers have recently investigated whether H1R antagonists inhibit tumor progression. Terfenadine, a selective antagonist of H1R, not only inhibited cell proliferation and colony formation but also significantly reduced the cell migration and invasion of SNU-368 cells (an HCC cell line), confirming the involvement of H1R in tumor growth and metastasis [126]. Patricia et al. used terfenadine to block H1R in basal BC and BC resistant to HER2-targeted therapy and found that the blockade of H1R suppressed cell proliferation through sub-G0 cell accumulation and triggered activation of ERK signaling to initiate apoptotic cell death. Moreover, terfenadine therapy reduced the growth of basal and trastuzumab-resistant BC cells in vivo [129]. Other H1R antagonists such as chlorpheniramine and diphenhydramine were also used to examine the effect of H1R blockade on tumor progression in many different tumor cell types. These include BC (MDA-MB-231 and MCF-7) [130], MM (A375, B16F10, HT144, and HSs294T) [131,132,133], leukemia (CCRF-CEM and Jurkat) [134], OC (OVCAR-3, UWB1-289, and OCV-316) [135], and prostate cancer (PC-3 and DU-145) [136] cell lines. In all of these cells, H1R antagonists reduced cell proliferation and induced cell apoptosis, rendering them attractive and promising to improve the therapeutic efficacy of cancer treatment.

In agreement with these results using various types of cancer cell lines, in vivo studies also demonstrated the inhibitory effects of H1R antagonists on tumor progression. Terfenadine treatment markedly inhibited the growth of xenograft tumors bearing SNU-368 cells and significantly inhibited lung metastasis [126]. Moreover, the antimelanoma activity of H1R inhibition by terfenadine or diphenhydramine in a mouse model of xenografts using B16F10 and human A375 MM cell lines was confirmed, showing dramatic decreases in tumor growth [32,133]. It is generally known that acquired resistance to traditional chemotherapy with long-term administration further leads to tumor recurrence and relapse [137]. It has been reported, both in vitro and in vivo, that terfenadine inhibits the growth, migration, and invasion of resistant cells, augmenting the effect of epirubicin in the treatment of advanced NSCLC. Therefore, terfenadine or its derivatives may serve as a promising approach to impede the development of resistance to chemotherapy in patients with advanced NSCLC [138].

All of these findings, along with their known safety profiles as medicines in current use, have triggered human clinical trials with H1R antagonists. Indeed, several studies have been published showing improved OS of cancer patients taking H1R antagonists: MM [139] and BC [140] with the use of loratadine and desloratadine, and NSCLC [141] and OC [135] with the use of some cationic amphiphilic antihistamine drugs (CAD). Overall, these in vitro and in vivo studies, as well as several human trials, have revealed a substantial relationship between cancer regression and H1R antagonists, supporting the potential use of H1R antagonists in combination with traditional therapy to improve their therapeutic efficacy and/or impede the development of resistance to therapy. The in vitro and in vivo effects of H1R antagonists on tumor progression in different cancer types are summarized in Table 2.

#### 4.2.2. Histamine H2 Receptor

Depending on the subtypes of HR activated by histamine in mammalian cells, the resulting physiological response may be different. H2R signaling regulates gastric acid secretion in the stomach, and H2R antagonists have originally been developed for the treatment of peptic ulcers [142]. Following the widespread use of these drugs, anecdotal reports have been presented showing that H2R antagonists may alleviate malignant stomach ulcers and reduce the incidence of other malignancies, including CRC and BC [143,144,145,146]. These findings prompted a series of scientific studies to determine the nature and characteristics of the effects of H2R antagonists on cancer.

Several mechanisms by which H2R antagonists may counteract tumor progression have been proposed. The first mechanism is enhancement of the host’s immune function. As mentioned above, histamine, through H2R, disrupts the balance between TH1 and TH2 cells and suppresses CD8+ CTL; thereby, it inhibits antitumor activity. Cimetidine reversed these effects by increasing the antigen-presenting capacity of DCs to T cells and CTL CD8+ in CRC patients [142,147]. In addition, cimetidine significantly increased the levels of several cytokines such as IFN-α, IL-2, IL-12, and IL-15 [148,149,150,151,152,153]. These cytokines are known to inhibit tumor proliferation in vivo and in vitro [154,155,156,157]. Moreover, H2R antagonists promote tumor-infiltrating lymphocyte (TILs) responses to the tumor site [21,158,159]. The presence of TILs is a prognostic marker for improved OS as well as a better response to chemotherapy [160]. Histamine is commonly detected at higher levels in the TME, which prevents TILs from recognizing tumor antigens and invading. Both cimetidine and famotidine augmented TILs in patients with CRC in randomized, double-blind, and placebo-controlled studies [154,161,162] and ranitidine was also shown to improve the antitumor activity of NK cells in a study using the peripheral blood from patients with liver metastasis of CRC [163]. Similar results were achieved with the preoperative applications of cimetidine on patients with gastric cancer (GC) [164] and famotidine on BC patients [165].

The direct inhibition by H2R antagonists of histamine-induced tumor cell proliferation may be another potential mechanism. As higher levels of histamine are distributed widely throughout the intestinal tract, H2R is also found in various gastrointestinal tumors, including esophageal squamous cell carcinomas (ESCC), CRC, GC, HCC, cholangiocarcinoma (CCA), and pancreatic cancer (PC) [166]. Several studies have reported that the proliferation of several gastrointestinal cancers is mediated by histamine via H2R, and this effect can be reversed by H2R antagonists [166]. Cimetidine exhibited antiproliferative and/or pro-apoptotic effects in human CRC [167], GC [168], and CCA cell lines [169], as well as antitumor growth in xenograft mice bearing these cells. Reductions of cellular proliferative markers such as phosphorylation of Akt and/or the induction of cellular apoptotic markers such as caspase-dependent apoptosis and Bax/Bcl-2 ratio are involved in the antitumor actions of cimetidine. In other studies conducted in HCC cell lines, cimetidine disrupted the epidermal growth factor (EGF)-induced autophosphorylation of EGF receptors and its downstream effectors by reducing the cAMP concentration, resulting in suppression of EGF-induced proliferation and migration [170]. In agreement with these preclinical studies, clinical trials using H2R antagonists (ranitidine and cimetidine) showed significant increases in OS when they were used as an adjuvant treatment for patients undergoing curative resection of CRC [159,171] or head and neck squamous cell carcinoma [172]. Nevertheless, the administration of H2R antagonists failed to reduce tumor growth in some other cancers. Ranitidine did not exhibit any antitumor effect in B16F10 MM, LLC1 lung cancer, and EF4 lymphoma models [173]. In addition, the administration of ranitidine had a 2.2-fold increased risk of ductal BC, while concurrent uses of two other H2R antagonists, cimetidine and famotidine, had no relationship [174]. According to these observations, the direct inhibition of tumor growth by H2R antagonists seems to be inconclusive.

**Figure 3:**
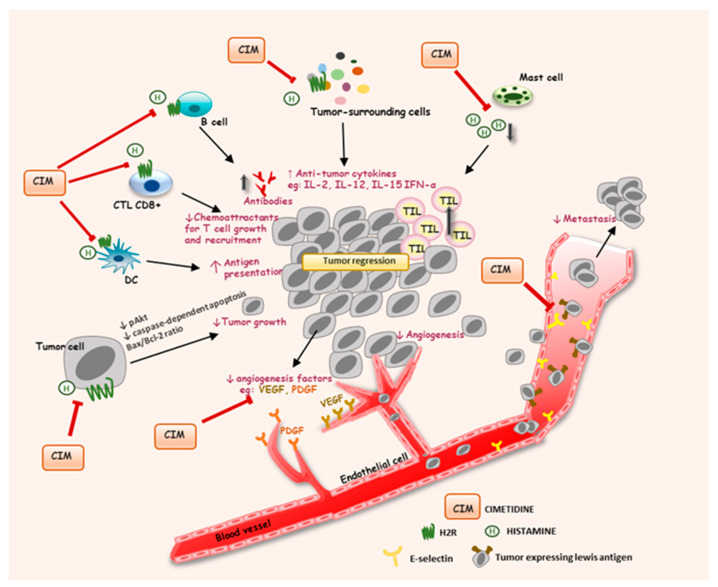
Potential action mechanisms underlying the antitumor effects of cimetidine. See the text for detailed mechanisms by which cimetidine exhibits antitumor activities in many different types of cancer. MC: mast cell; CTL CD8+: cytotoxic T lymphocyte; TILs: tumor-infiltrating lymphocytes; DC: dendritic cell; VEGF: vascular epithelial growth factor; PDGF: platelet-derived endothelial growth factor; H2R: histamine H2 receptor.

A third mechanism mediating the antitumor activity of H2R antagonists may involve their actions in angiogenetic and metastatic pathways. Cimetidine seems to counteract the cell surface expression of E-selectin, an adhesion molecule of endothelial cells. E-selectin plays a key role in the binding of endothelial cells to cancer cells by means of a specific interaction with sialyl-Lewis antigens [175]. This process is suspected to be associated with tumor angiogenesis and hematogenous metastasis of HepG2 cells [176]. In another in vitro work, cimetidine dose-dependently inhibited the adhesion of human CRC cells to activated human umbilical vein endothelial cells [177]. Furthermore, cimetidine also exhibited suppression of blood-borne liver metastases in xenograft mice bearing CRC cells [177]. These effects of cimetidine also occurred in GC and HCC cells, and it completely prevented the onset of metastasis to the other organs in animal models [176,178]. Of note, this ability appears to be unique to cimetidine, suggesting its promising therapeutic potential in cancer by disrupting the function of E-selectin on the endothelial cell surface. Besides, H2R antagonists attenuate angiogenesis by reducing VEGF. Tomita et al. reported that two H2R antagonists, namely roxatidine and cimetidine, markedly inhibited the levels of VEGF in syngeneic colon cancer (Colon 38)-implanted mice, suppressing the growth of CRC implants [179]. Additional study of CRC reported that histamine significantly augmented cell proliferation and VEGF production via a COX-2 cascade, and administration of cimetidine notably inhibited this effect [180]. Cimetidine also revealed antitumor activity via inhibiting several angiogenesis factors such as VEGF and platelet-derived endothelial growth factor in chemically-induced bladder carcinogenesis in mice and rats [181]. The potential action mechanisms underlying the antitumor effects of cimetidine are illustrated in Figure 3.

#### 4.2.3. Histamine H3 Receptor

H3R is mainly localized in the CNS and has been described as a presynaptic autoreceptor, controlling the release of neurotransmitters from histaminergic neurons. Targeting H3R has focused on neuronal diseases such as cognitive impairment, schizophrenia, epilepsy, sleep/wake disorders, and neuropathic pain [182]. Evidence indicating a potential relationship between H3R and cancer is scarce, and key questions remain elusive. Recent studies, however, have demonstrated that H3R expression is upregulated in several types of cancer, including GC, HCC, BC, lung cancer, and MM cancer cells [12,183,184], suggesting that H3R appears to diversify their signaling effects in these cell lines. After the publication of these studies, more attention has been paid to in vitro and in vivo studies to elaborate on the involvement of H3R in cancer.

Tanaka et al. examined the effects of H1R~H3R antagonists on the inflammation-associated colorectal carcinogenesis induced by azoxymethane in male ICR mice. While the H1R antagonist terfenadine did not affect azoxymethane-induced carcinogenesis, cimetidine (H2R antagonist) and clobenpropit (H3R antagonist) remarkably reduced the multiplicity of colonic adenocarcinoma [185]. In another study, an H3R agonist (imetit) and clobenpropit were employed to systematically explore the role of H3R in HCC growth and metastasis in vivo and in vitro. Upregulation of H3R has been demonstrated to facilitate tumor progression through induction of lamellipodia. These effects are mimicked by imetit and reversed by clobenpropit, indicating that H3R plays an important role in the growth and metastasis of HCC cells [183]. Similar findings were observed in the malignant lesions biopsied from BC patients and MDA-MB-231 breast cancer cells [13]. Because the level of H3R expression was significantly higher in carcinoma tissues, the overexpression of H3R is highly correlated with increasing levels of histamine production, cell proliferation, and migration of MDA-MB-231 cells [13]. In fact, clobenpropit and OUP-186, another H3R antagonist, were shown to effectively suppress the proliferation and migration of MDA-MB-231 and/or MCF-7 cells [13,36].

The involvement of H3R in the growth and metastasis of NSCLC was recently revealed by Zhao et al. [184]. Based on the observation that high expression levels of H3R were correlated with poor OS in NSCLC patients, they employed ciproxifan (an H3R antagonist) to test its antitumor activities and found that ciproxifan exerted moderate and concentration-dependent inhibition of cell proliferation and induced apoptosis in NSCLC cells. Their migration and invasion were also inhibited by ciproxifan, which was mediated by EMT inhibition via reducing phosphorylation of the PI3K/Akt/mTOR and MEK/ERK pathways [184]. These results were also confirmed in an in vivo study using nude mice bearing H1975 or A549 cell xenografts. Ciproxifan increased E-cadherin and ZO-1 expression and decreased fibronectin expression in tumor tissues, thereby reducing the tumor growth of NSCLC [184]. In contrast, however, (R)-(α)-(−)-methylhistamine (RAMH), an H3R agonist, decreased the cell proliferation of CCA in vitro and inhibited the tumor growth of xenograft nude mice with enhanced apoptosis via these signaling pathways [186].

Taken together, H3R expression is upregulated in several types of cancer. However, the functional role of H3R in tumor progression is still inconclusive. While RAMH (an H3R agonist) inhibited cell proliferation and tumor growth in CCA, various H3R antagonists, including clobenpropit, OUP-186, JNJ10181457, and ciproxifan, have been demonstrated to inhibit cell proliferation and tumor growth in different cell lines, as well as in vivo xenograft animal models of CRC, HCC, BC, and NSCLC. Based on these observations, H3R may be a potential therapeutic target for these cancer types.

#### 4.2.4. Histamine H4 Receptor

The identification of H4R, the last discovered subtype of HR, has helped broaden our knowledge concerning the roles of histamine in immunomodulation, leading to subsequent attention directed toward the development of various H4R ligands [8,187,188]. The genomic data of The Cancer Genome Atlas (TCGA) indicate that H4R is expressed in several cancer cell lines and in biopsy tissues from BC, GI, ESCC, MM, leukemia and lymphoma, kidney, and lung cancer patients [11,35]. This raises a question concening the functions of H4R and H4R modulators in cancer. In this section, we present current understandings of H4R in cancer progression and discuss the therapeutic potential of H4R agonists in cancer treatment. In addition, the prognostic values of H4R expression in different types of cancer are discussed.

According to the TCGA database, H4R gene expression is remarkably higher in the primary tumors of CCA, HCC, ESCC, and kidney renal clear cell cancer [35]. Patients with increased expression of H4R protein in tumor cells have a larger tumor size and a greater number of metastases than those with less H4R expression, suggesting that H4R levels may serve as a prognostic marker for these cancers. Conversely, down-regulated expression of H4R is observed in BC, CRC, bladder urothelial carcinoma, uterine corpus endometrial carcinoma, and lung cancer. Hence, the reduced H4R gene expression in these types of cancers may also be considered as a predictive factor [35]. Interestingly, the OS rate is reduced for kidney renal clear cell carcinoma patients with higher expression of H4R, indicating an inverse correlation of OS and H4R gene expression in this cancer type [35]. However, it is very difficult to confirm the connections between H4R expression levels and OS in other types of cancer due to limited information.

Tumor cells manifest characteristics that allow cells to proliferate abnormally and to survive beyond their normal life span [189]. Cancer therapy can be defined as cytotoxic agents causing cell death when the basal levels of regeneration and proliferation are higher than their normal level. As the function of H4R in tumor progression has been demonstrated, several H4R ligands, particularly H4R agonists, have received more attention as promising drugs in cancer treatment [16,35,190,191,192,193,194]. Indeed, H4R stimulation by selective H4R agonists not only significantly suppressed cell proliferation in BC [37,195], MM [190], ESCC [196], CRC [197], GC [198], and PC [199], but also induced cell cycle arrest (G0/G1 phase) and increased apoptotic cell death and senescence. In contrast, H4R antagonists totally blocked the antiproliferative effects of H4R agonists. Similar results were also observed in the xenograft models of BC, MM, ESCC, and PC using MDA-MB-231, M1/15, TE-2, and Panc-1 cells, respectively, in nude mice [37,196,199,200]. There are several mechanisms underlying the inhibitory effects of H4R agonists on cell proliferation in these cancer types. The activation of H4R consistently inhibits cAMP production and subsequently reduces cAMP-driven gene transcription. The use of a selective H4R agonist leads to a reduction of cell proliferation via this pathway in human CRC [197] and MM [200]. Other mechanisms have been hypothesized in ESCC, in which H4R activation causes inhibition of TGF-β1 signaling via the metabolism pathway (via acetyl-coenzyme A synthetase 2) or the MAP kinase-mediated non-metabolism pathway [196].

H4R is also involved in tumor metastasis in several cancer types [199,201,202,203]. EMT alters cell-cell adhesion, morphology, and the migratory and invasive capacities of tumor cells, resulting in tumor metastasis [204]. During EMT, the extracellular matrix is broken down by the upregulated MMPs in the TME in close association with down-regulated epithelial markers and overexpressed mesenchymal markers [205]. Clobenpropit, acting at H4R as an agonist, suppresses the metastasis of CCA and PC by disrupting EMT and altering morphological invasive development [199,202]. In line with this phenotype change, 4-methylhistamine, another H4R agonist, is also implicated in EMT suppression through inhibiting the TGF-β1 signaling pathway in NSCLC, which is reversed by JNJ7777120 [203]. JNJ7777120 is known to act as a specific antagonist at H4R, as shown in Table 1. Intriguingly, however, JNJ7777120 has been demonstrated to recruit β-arrestin to H4R, without activating G proteins [206]. Thus, it will be interesting to further elucidate the signaling pathway(s) mediating the effect of JNJ7777120 to reverse EMT suppression in these types of cancer. Besides, the H4R agonist increased the expression of epithelial marker E-cadherin and decreased the expression of mesenchymal markers fibronectin and vimentin in four different NSCLC cell lines. In addition, several MMPs, including MMP2 and MMP9, were downregulated.

In the immune system, H4R is preferentially expressed in the immune cells, such as MCs, T cells, DCs, and NK cells (Table 1). It is well-recognized that H4R plays crucial roles in these cells during inflammatory processes. The immunomodulatory function of H4R is also of significance in cancer biology. H4R-KO mice were used to elucidate the participation of H4R in antitumor immunity in BC. The tumor-bearing KO mice had better survival compared to wild-type mice. The KO mice exhibited decreased tumor size and weight, reduced number of lung metastases and CD4+ TILs, and increased infiltration of CD8+ T cells and NK cells in the tumor-draining lymph nodes in BC [193,207]. Afterward, the potential use of an H4R ligand in immunocompetent hosts has been validated in a model of triple-negative BC. Histamine treatment reduced tumor growth and increased apoptosis in 4T1 tumor-bearing mice, together with a higher cytotoxic infiltration of lymphocytes [208]. These findings support the immunomodulatory roles of H4R in antitumor functionality, highlighting the therapeutic potential of H4R ligands as adjuvants to cancer therapy.

It has been reported that H4R shows genetic and post-transcriptional variations, and the polymorphisms of the H4R gene may alter its expression and function. Indeed, the pathophysiological functions of H4R are affected by several genetic single-nucleotide polymorphisms that were subsequently associated with many immunological diseases such as asthma or atopic dermatitis [209,210]. Similarly, a case-control study in Chinese Han BC patients found that H4R variants of rs623590, rs11662595, and rs1421125 are significantly associated with the risk and malignant degree of BC [211]. Subsequently, in vitro and in vivo studies using A549 cells transfected with either wild-type or rs11662595 mutated H4R clones showed that rs11662595 significantly decreased the ability of H4R to activate the Gi protein, resulting in a facilitation of the EMT progress, cell proliferation, and invasion. Consistent with these results, a prospective cohort study in NSCLC patients further proved that the rs11662595 variant was responsible for H4R dysfunction and EMT progress. Therefore, rs11662595 is a loss-of-function polymorphism, providing a promising biomarker for the prognosis and therapy of NSCLC [212]. H4R isoforms are the results of alternative splicing. Despite shorter isoforms being expressed in human cells, they could not bind to H4R ligands and induce signaling or constitutive activities. They presumably produced a dominant-negative effect on H4R full-length [24,213]. Interestingly, H4R gene alterations are exhibited to different degrees depending on the cancer types. It is worthwhile to further investigate the genomic alteration of H4R because it may provide useful knowledge for the development of potential drugs and new insight into promising biomarkers for cancer.

In brief, the activation of H4R by specific ligands such as clobenpropit and 4-methylhistamine inhibits tumor progression in many different types of cancer. Multiple mechanisms, such as immunomodulatory function in the TME, are involved in the H4R-mediated antitumor action. Data from independent research groups demonstrated the promising therapeutic potentials of H4R ligands in BC, MM, CRC, ESCC, PC, and NSCLC. The in vitro and in vivo effects of H4R agonists in different cancer types are summarized in Table 3. It is important to highlight that the contribution of genetic and post-transcriptional variations of H4R is of significance for therapeutic outcomes. Correlations of some H4R polymorphisms with malignancies have been reported. Taken together, the targeting of H4R may be a promising approach to improve therapeutic outcomes in cancer treatment. Thus, H4R ligands would offer a novel therapeutic potential as adjuvants for various types of cancer, including BC and NSCLC. However, further preclinical and clinical studies are warranted before H4R ligands can be used in clinical settings.

## 5. Conclusions

Despite the enormous advances in cancer research over the last few decades, the therapeutic outcomes of the current treatments are not satisfactory. Various attempts have been made to identify novel potential targets for cancer treatment. High concentrations of histamine, along with the overexpression of HRs, are manifested in many different types of tumor cells, strongly suggesting their involvement in cancer biology. In this review, we summarized the pathophysiological roles of histamine and their receptors in cancer progression and described their implications and perspectives as potential molecular targets for cancer treatment. We also presented potential prognostic values of the HR subtypes and discussed the novel therapeutic approaches of selective HR ligands in cancer.

Histamine exerts multidimensional activities through interactions with its cognate receptors expressed on many different types of immune cells, including MCs, MOs, DCs, B and T cells, and MDSCs. Histamine recruits these cells into the TME and regulates their proliferation and differentiation to exhibit either pro- or antitumor effects.

Based on genomic analysis using the PrognoScan database, the expression of the H1R gene is significantly correlated with cancer prognosis. In agreement with this finding, the activation of H1R promotes cell growth, migration, and invasion of tumor cells, including several types of gastrointestinal cancers. In contrast, H1R antagonists, such as terfenadine and chlorpheniramine, were found to reduce the proliferation of tumor cells and induce apoptosis, highlighting H1R as an attractive and promising target in cancer treatment. Besides, the activation of H2R was shown to decrease the host’s immune function through disrupting the TH1 and TH2 balance and suppressing CD8+ CTL cells. It is worth noting that cimetidine, a selective H2R antagonist, reversed the H2R-mediated suppression of immune function via multiple action mechanisms. Angiogenesis and metastatic pathways of tumor cells were also inhibited by cimetidine. Although the functional role of H3R in tumor progression is still inconclusive, the stimulation of H4R suppresses cell proliferation and induces cell cycle arrest and apoptotic cell death in various types of cancer cells, including BC and CRC. Indeed, clobenpropit and several other H4R agonists have been demonstrated to suppress the metastasis of CCA and PC by disrupting EMT and altering morphological changes for invasion. Furthermore, CCA and HCC patients with increased expression of H4R in tumor cells had a larger tumor size and more metastases, suggesting the expression level of H4R as a potential prognostic marker for these types of cancer.

Taken together, substantial evidence from in vitro and in vivo studies, as well as from several human trials, indicates a strong relationship between cancer regression and various HR ligands, including H1R antagonists, H2R antagonists, and H4R agonists. Therefore, these HR subtypes are promising molecular targets for cancer treatment, and HR ligands may improve therapeutic efficacy, offering novel therapeutic application alone or in combination with conventional therapy. However, due to the complexity of the pathophysiological roles of histamine and HRs in cancer biology, additional studies are required to fully clarify the specific roles of histamine and its receptor subtypes in many different types of cancer. As in other cells, cancer cells may co-express several types of HRs. Therefore, development of highly specific pharmacological tools and well-designed knockout studies may be useful to decipher the specific roles of the respective HR subtypes in cancer cells.

## Figures and Tables

**Table 1:** Distribution of HR subtypes and their specific agonists and antagonists [9,11,22,23,24,27,28,30,32,33,34,35,36,37].

HR Subtype	Distribution	Agonist	Antagonist/Inverse Agonist
H1R	B cells, cancer cells, chondrocyte, dendritic cells, endothelial cells, eosinophils, hepatocytes, mast cells, monocytes, neutrophils, nerve cells, smooth muscle, and T cells	Histaprofiden, methylhistaprodifen, and suprahistaprodifen	Astemizole, cetirizine, chlorpheniramine, cyproheptadine, diphenhydramine, fexofenadine, loratadine, mepyramine, pheniramine, pyrilamine, terfenadine, and triprolidine
H2R	B cells, cancer cells, chondrocyte, dendritic cells, endothelial cells, eosinophils, epithelial cells, gastric parietal cells, hepatocytes, mast cells, monocytes, neutrophils, nerve cells, smooth muscle, and T cells	Amthamine, dimaprit, and impromidine	Burimamide, cimetidine, famotidine, lafutidine, ranitidine, and tiotidine
H3R	Cancer cells, eosinophils, histaminergic neurons, and monocytes	Imetit, immepip, immethridine, and R-α-(-)methylhistamine	Clobenpropit, ciproxifan, JNJ5207852, JNJ10181457, OUP-186, pitolisant, and thioperamide
H4R	Basophils, cancer cells, dendritic cells, hematopoietic cells, hepatocytes, leukocytes, mast cells, monocytes, neutrophils, and T cells	Clobenpropit, JNJ28610244, 4-Methylhistamine, ST-1006, VUF 6884, and VUF 8430	A-940894, A-987306, JNJ7777120, JNJ10191584, JNJ39758979, thioperamide, VUF 6002, and ZPL-3893787

HR: histamine receptor; H1R: histamine H1 receptor; H2R: histamine H2 receptor; H3R: histamine H3 receptor; H4R: histamine H4 receptor.

**Table 2:** In vitro and in vivo effects of H1R antagonists on tumor progression in different cancer types.

Cancer Type	Experimental Models	H1R Antagonists	Effects	References
HCC	SNU-368	Terfenadine	↓ proliferation ↓ migration ↓ invasion	[126]
	SNU-368 xenograft mice		↓ tumor growth ↓ metastasis	
BC	Basal and HER2-targeted therapy-resistant cells	Terfenadine	↓ proliferation ↑ apoptosis	[129]
	MDA-MB-231 MCF-7	Chlorpheniramine	↓ cell number	[130]
	MDA-MB-231 T-47D xenograft mice	Terfenadine	↓ tumor growth	[129]
	Human	Desloratadine Loratadine	↑ survival rate	[140]
NSCLC	A549	Terfinadine	↓ migration ↓ invasion	[138]
	A549 xenograft mice		↓ tumor growth ↓ metastasis	
	NSCLC	CAD	↓ mortality	[141]
MM	A375 HT144 HSs294T	Terfenadine	↑ apoptosis	[131]
	A2058 A375	Diphenhydramine	↑ apoptosis	[133]
	B16F10 xenograft mice		↓ tumor growth ↑ survival time	
	B16F10 A375 syngeneic mice	Terfenadine	↓ tumor growth	[32]
	Human	Desloratadine Loratadine	↑ survival rate	[139]
Leukemia	CCRF-CEM Jurkat	Diphenhydramine	↑ apoptosis	[134]
OC	OVCAR-3 UWB1-289 OCV-316	CAD	↑ cell death	[135]
	Human	CAD	↓ cancer mortality	
Prostate cancer	PC-3 DU-145	Terfenadine	↓ proliferation ↑ apoptosis	[136]

↑: increase; ↓: decrease; HCC: hepatocellular carcinoma; BC: breast cancer; NSCLC: non-small cell lung cancer; MM: malignant melanoma; OC: ovarian cancer; CAD: cationic amphiphilic antihistamines drugs.

**Table 3:** In vitro and in vivo effects of H4R agonists on tumor progression in different cancer types.

Cancer Type	Experimental Models	H4R Agonists	Effects	References
BC	MDA-MB-231	Histamine Clozapine JNJ28610244	↓ proliferation ↑ apoptosis	[37]
	MDA-MB-231 MCF-7	Clobenpropit VUF 8430	↓ proliferation ↑ apoptosis ↑ senescence	[195]
	MDA-MB-231 xenograft mice	Histamine Clozapine JNJ28610244	↓ tumor growth ↓ angiogenesis ↓ metastasis	[37]
	4T1 syngeneic mice	Histamine	↓ tumor growth	[208]
MM	WM35 M1/15	Clobenpropit VUF 8430	↓ proliferation ↑ senescence	[190]
	M1/15 xenograft mice	Clozapine	↓ tumor growth ↑ survival time	[200]
	1205Lu xenograft mice	Histamine	↓ metastasis	[201]
	1205Lu	Histamine plus ionizing radiation	↓ proliferation ↑ apoptosis	
ESCC	TE-2	4-Methylhistamine	↓ proliferation ↓ invasion ↓ metastasis	[196]
	TE-2 xenograft mice	4-Methylhistamine	↓ tumor growth ↑ survival time	
CRC	Colo-320 Lovo	Clozapine	↓proliferation ↑ cell cycle arrest ↑ apoptosis	[197]
GC	AGS	Clobenpropit 4-Methylhistamine	↓ proliferation ↑ cell cycle arrest	[198]
PC	Panc-1	Clobenpropit	↑ apoptosis ↓ migration	[199]
	Panc-1 xenograft mice	Clobenpropit	↓ tumor growth	
CCA	Mz-ChA-1 SG231 HuH-28 TFK-1 HuCCT-1 CCLP1	Clobenpropit	↓ proliferation ↑ apoptosis	[202]
	Mz-ChA-1	Clobenpropit	↓ proliferation ↓ invasion, ↓ migration	
NSCLC	H157 H460 A549 H322	4-Methylhistamine	↓ invasion ↓ metastasis	[203]
	A549 xenograft mice	4-Methylhistamine	↓ tumor growth ↑ survival time	

↑: increase; ↓: decrease; BC: breast cancer; MM: malignant melanoma; ESCC: esophageal squamous cell carcinoma: NSCLC; CRC: colorectal cancer; GC: gastric cancer; PC: pancreatic cancer; CCA: cholangiocarcinoma; NSCLC: non-small cell lung cancer.
